# Drivers and collaborative governance of public health emergency response in the context of digital city

**DOI:** 10.3389/fpubh.2024.1417490

**Published:** 2024-07-18

**Authors:** Yang Chen, Yu Yu

**Affiliations:** ^1^School of Management Engineering, Xuzhou University of Technology, Xuzhou, China; ^2^School of Economics and Management, China University of Mining and Technology, Xuzhou, China

**Keywords:** public health, driving factors, collaborative governance, temporal and spatial characteristics, grounded theory, emergency management

## Abstract

**Introduction:**

With the frequent occurrence of public health events, the government inevitably makes many mistakes in emergency management. In modern emergency management, it is particularly important to promote the diversification of emergency management subjects and improve the government’s emergency management ability.

**Methods:**

In order to make up for the deficiency of government’s participation in public health emergency management, this paper analyzes the driving factors and driving effects of enterprises’ participation in public health emergency response under the background of digital city. A fully explained structural model is used to analyze the relationship between the different drivers. In addition, the spatial and temporal distribution characteristics of public health events were analyzed through spatial auto-correlation. On this basis, the government cooperative governance strategy is discussed.

**Results and discussion:**

The results show that in the context of digital cities, there are 14 driving factors for enterprises to participate in public health emergency response. The most important factors are the company’s own development needs, relative technical advantages and so on. The driving efficiency is mainly concentrated in three aspects: psychology, resources and structure. Public health events have periodicity in time distribution and regional differences in spatial distribution. The significance of this study is to help the government improve the emergency management ability from different angles.

## 1 Introduction

Public health emergencies (PHE) have the characteristics of suddenness and unpredictability, which also pose challenges to the life and property. To cope with the impact of sudden public health incidents on the population, China established an emergency management system in 2009 (1, 2). However, faced with increasingly complex and ever-changing PHE, the shortcomings of government monopolized emergency response are gradually exposed. In order to effectively prevent emergency failures, the government has made it inevitable to involve multiple social forces in emergency management. The participation of multiple stakeholders will enhance the overall effectiveness of emergency management and to some extent reduce economic losses. Enterprises, as representatives of social forces, play a very important role in emergency response to emergencies. From multiple domestic and international emergency response practices, enterprises have long become an indispensable and important entity in the emergency response process. In order to enhance the government’s governance capacity, some experts have conducted research on the participation of social forces and enterprises in emergency response. Some studies have also analyzed the spatiotemporal characteristics of PHE from the epidemiology and geography, in order to understand the differences in public health emergencies in different regions and take targeted governance measures (3). However, these studies also have certain shortcomings, such as the scattered content and weak theoretical basis of research on enterprise participation in emergency response (4). Digital technology can help governments play an important role in preventing and controlling pandemics. Based on the spatiotemporal characteristics of PHE, the government should better formulate relevant prevention and control measures, enhancing the emergency management capabilities (5). Collaborative governance, as a governance arrangement, can effectively enhance the government’s emergency management capabilities. In order to design a medical data open governance model for major public health emergencies, Li et al. (6) researchers used literature review and network survey methods to analyze the current research status and existing obstacles of the medical data open model, and proposed a governance plan. Lanford et al. (7) experts have designed a new cross departmental coordination framework for organizational collaboration across social services, public health, and healthcare. The core of this framework is to develop collaborative systems in four core areas, promoting the effectiveness and sustainability of collaboration (7). Based on these issues, the grounded theory is proposed to analyze the driving factors and driving effectiveness of enterprise participation in emergency response. The spatial autocorrelation analysis and spatiotemporal scanning statistical methods are used to analyze the spatiotemporal characteristics of sudden public health events. Based on this analysis, government collaborative governance measures are designed. There are two innovative points in the research. One is to improve the government’s emergency management capabilities from the participation of enterprises in emergency response and the spatiotemporal characteristics of PHE. The other is a combination of grounded theory and fully interpretive structural models. The research aims to enhance the government’s ability to participate in emergency management of PHE, enhance society’s resilience to PHE, and further safeguard the safety of life and property.

The contribution of the research is to effectively combine public health collaborative governance with the spatiotemporal distribution of sudden PHE, identify the driving factors for enterprise participation in sudden PHE, and improve the overall effectiveness of social emergency management. The research approach is applicable to the collaborative governance and driving factors of other specific types of PHE. The spatial autocorrelation analysis and spatiotemporal scanning statistical methods used in the study are not only applicable to the spatiotemporal distribution analysis of PHE, but can also be applied in other fields, such as the coupling relationship between technological innovation and regional economic development, and the coupling relationship between human geography and economic development.

The study is divided into four parts. The first part is a literature review related to PHE. The second part is the specific design of the used method. The third part is the analysis of results using different methods. The fourth part is the conclusion, shortcomings, and future work of the study.

## 2 Related works

In recent years, the frequency of PHE has gradually increased, bringing many serious injuries to people. At the same time, people realize that relying solely on the government for emergency response to PHE is not enough. Therefore, more experts are conducting research in the PHE. The study first reviewed the literature related to the groups and communities involved in PHE, followed by a review of the staff involved in PHE, and finally a review of other aspects involved in PHE. Kim et al. (8) selected the Middle East Respiratory Syndrome Coronavirus in South Korea as a research case to analyze the effectiveness of emergency response teams in PHE. The impact of unexpected groups on communication and coordination between two organizations was analyzed. The research results showed that this group promoted information exchange between organizations, but could not promote resource coordination (8). Wang et al. (9) used a quantitative case study method to evaluate community health services under sudden PHE. In addition, geographic information systems were used to obtain data, such as the LocalSpace Viewer. The research results indicated that there were differences in community health services between urban and rural areas. Moreover, the sanitation facilities at the administrative boundary were limited (9). Lanford et al. (7) designed a cross departmental coordination framework to address the lack of collaboration among organizations spanning social services, public health, and healthcare. Collaborative systems were developed in core areas. In addition, the study also designed guiding questions. There was a significant gap in resource level and experience accumulation between organizations and individuals engaged in cooperation (7). Ferguson et al. (10) developed an evidence-based measurement tool to assess the emergency preparedness of communities. It measured the emergency preparedness of ordinary households in radiation emergencies. In addition, the study also used community assessment methods for PHE response to collect data. The research results indicated that this method collected information and data related to radiation preparedness, enhancing the ability of ordinary households to respond to radiation emergencies (10).

Li et al. (11) obtained information from multiple databases through literature review to cultivate the emergency management ability of leaders in PHE. This mainly involved experiential learning, technology assisted decision support tools, leadership, adaptability, and character. The research results indicated that these training contents enhanced the emergency management ability of leaders (11). Chiang et al. (12) conducted a qualitative study to determine the factors that motivated federal public health workers to continue working on-site during emergencies. An interview was conducted with staff from a certain Centers for Disease Control and Prevention in the United States. In addition, the study also used grounded theory to process data. The research results showed that identifying and introducing incentive factors could enhance the willingness of federal public health workers to participate in emergency events (12).

Leeb et al. (13) proposed a new coordination framework and strategy to improve nursing coordination and communication in PHE. This framework involved the collection and transmission of key information, communication paths and transfer methods across touch points. It could be modified and adjusted. The research results indicated that this framework could effectively address the nursing coordination. Long term and multidisciplinary management could be achieved (13). Baines et al. (14) selected COVID-19 in 2020 as a research case to study the ethical adjustment problems that occurred during and after PHE. The ethical values supporting health services were sorted out and the impact of ethical challenges on healthcare personnel was analyzed. When facing sudden PHE, attention should be paid to public health ethics (14).

In summary, current research on PHE involves many sub areas, such as the participation of social forces in emergency response, which mainly focuses on participation modes and influencing factors, and the auxiliary effects of digital technology in emergency response. However, current research also has certain limitations, such as the relative lack of research on the participation of social forces in emergency management under the digital background, and the lack of relevant research on how digital technology drives enterprises to participate in emergency response. In addition, current research uses grounded theory, evidence-based methods, quantitative case study methods, and literature review methods. The differences between these studies are mainly reflected in their research content and methods. Some literature uses quantitative case study methods, while others use literature survey methods. The impact of these differences on research is not to be limited to a single method, but to leverage the advantages of different methods to better serve research objectives. Therefore, based on the grounded theory, the driving factors and driving effectiveness of enterprise participation in public health emergency response in the digital cities are analyzed. The spatiotemporal characteristics of sudden public health events are also analyzed based on spatial autocorrelation analysis and spatiotemporal scanning statistical methods. The incremental contribution of the study is to analyze the driving factors of enterprise entities participating in emergency response in the context of digitalization, enriching research in this field, especially in the context of rapid development of digitalization and information technology.

## 3 The analysis methods for identifying emergency response drivers and spatiotemporal distribution characteristics in the digital cities

To compensate for the lack of government involvement in public health emergency management, this study uses grounded theory to obtain influencing factors, and uses a fully explanatory structural model and a cross influence matrix multiplication model to analyze driving factors. These technologies can also be repeatedly applied to identify and analyze the influencing factors of other social forces, and the identification of enterprise driving factors can also be repeatedly applied to other events. To better compensate for the government’s shortcomings in emergency management and learn the temporal and spatial distribution characteristics, the spatial autocorrelation analysis and spatial econometric models are applied in the study. These methods can be reused in the coupling relationship between human geography and economic development, and the spatiotemporal characteristics of PHE can also be reused in other research on PHE governance.

### 3.1 Driver factors and effectiveness identification method for public health emergency response based on grounded theory and TISM model

**Figure 1:**
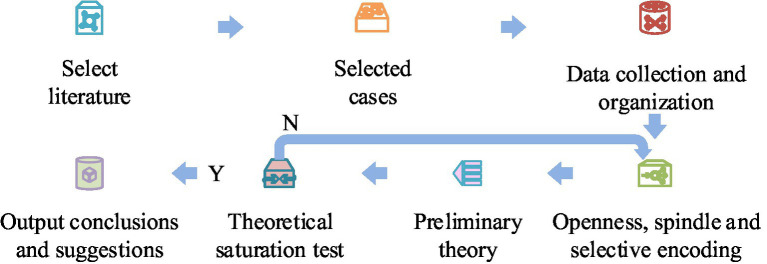
The main process of grounded theory.

In the context of digital cities, adopting technologies such as big data and artificial intelligence in emergency response to public health events has become one of the important ways to enhance emergency capabilities (15, 16). As representatives of social forces, enterprises possess a large amount of data and advanced digital technologies and products, such as health codes during the epidemic and digital online retail systems. In the context of digital cities, applying the digital technologies mastered by enterprises to emergency response to public health emergencies can improve the efficiency of emergency management (17). Therefore, it is particularly important to study the factors that drive enterprises to participate in emergency response to public health emergencies in the context of digital cities. To enhance the ability of enterprises to participate in public health emergency management and make up for the shortcomings of the government in emergency management, the driving factors and driving effectiveness of enterprise participation in emergency response in the digital cities are identified and analyzed based on the grounded theory. A Total Interpretative Structural Modeling (TISM) and a Matrices Impacts Crosses-Multiplication Appliance Classification (MICMAC) model are constructed to analyze the hierarchical structure of driving factors and the driving force-dependency between each factor. The TISM model can help to understand the interrelationships between the factors that promote enterprise participation in public health emergency response, and the MICMAC model can classify the driving factors for enterprise participation in public health emergency response. TISM is a decision-making tool that can explain task relationships between variables, such as binary or fuzzy relationships, through the TISM model. TISM can simplify the interpretation of complex systems into simple descriptions through explanatory matrices. Therefore, the study will use the TISM method to understand the interrelationships between the relevant factors that promote enterprise participation in emergency response. MICMAC analysis is often used to classify factors based on their ability to influence other factors, and can analyze driving and dependent factors. The output of TISM is the input for MICMAC analysis. Enterprises, as representatives of social forces, play a very important role in emergency response to emergencies. Therefore, analyzing the driving factors of enterprise participation in public health emergency response through the TISM model and MICMAC model can not only promote the improvement of social public health emergency response capabilities, but also improve the current governance system of public health emergency response, and promote enterprise participation in public health to become an effective supplement to government entities. The concept of grounded theory is a qualitative research method based on empirical data to establish a theory. This method is a bottom-up approach to establish substantive theories, and its main process is shown in Figure 1 (18).

**Figure 2:**
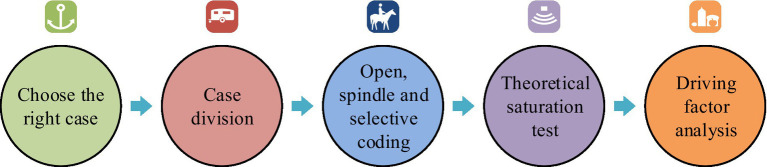
The main steps of identifying driving factors.

From Figure 1, it can be seen that grounded theory mainly includes seven processes. When applying grounded theory, it can be seen as four stages. Firstly, it is necessary to clarify the research question, which is the driving factors for enterprises to participate in PHE emergency response; Secondly, it is necessary to collect data on enterprise participation in emergency response through different channels. Afterwards, the collected data needs to be encoded, including open coding, spindle coding, and selective coding. Finally, construct a theoretical model for enterprise participation in emergency response. The main steps for identifying driving factors are shown in Figure 2.

**Figure 3:**
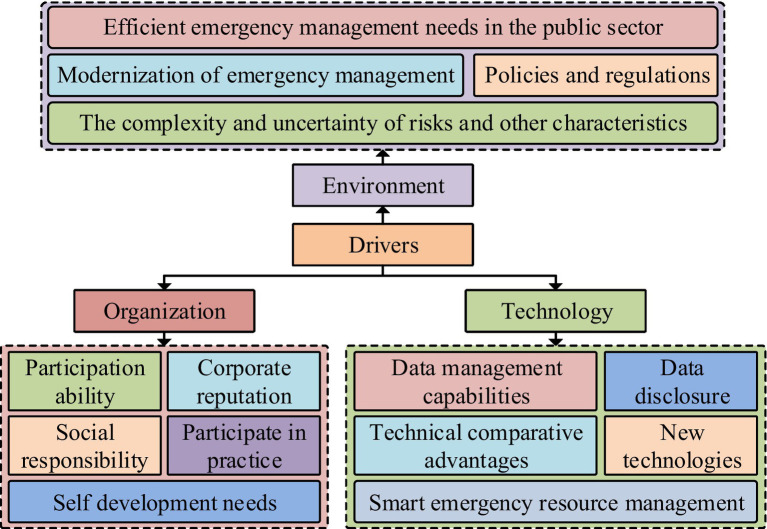
The driver model for enterprise participation in emergency response in the digital context.

From Figure 2, the first step in identifying the driving factors for enterprise participation in emergency response based on grounded theory is to select suitable cases. The selected channels include CNKI, Wanfang, government official websites and new media platforms, various search engines, etc. The second step is to divide the case into two parts, one for coding and the other for theoretical saturation testing. The third step is to encode the case in an open, spindle, and selective manner, and conduct a theoretical saturation test. The fourth step is to explain the identified driving factors. The main task of open coding is to extract initial concepts and categories. The main task of spindle encoding is to extract categories, integrate them into the scope of the core category, and organize the hidden relationships between different categories. The main task of selective coding is to merge substantive theories and verify the results of spindle coding. Theoretical saturation refers to the fact that if additional data and information do not provide the analyst with more novel initial concepts, categories, and relationships, it indicates that the current analysis results have good theoretical saturation. Otherwise, further data collection and analysis are needed. Among them, open coding includes seven steps, namely listing all answers, merging answers, sorting and categorizing, merging answers with similar meanings, counting the number of occurrences, forming a “coding table” and categorizing them one by one. For example, the normal implementation scope of emergency response includes the concepts of investigating resident traffic, isolating outsiders, and registering community entry and exit. The steps of spindle coding include checking the relationship between concept categories and various phenomena, verifying the above relationships, searching for the properties of concept categories, and testing the data of practical applications. For example, the main category of residents’ cooperation and participation includes the degree of cognitive and behavioral regulation of residents. Selective coding involves 5 steps, including creating storylines, connecting primary and secondary concept categories, developing faction types, verifying relationships between concept categories, and filling in categories that need to be supplemented or developed. Finally, the study selected the main category as the influencing factors of enterprise response to PHE. The storyline refers to the participation of enterprises in PHE, and there is an issue of execution deviation during the emergency prevention and preparation phase, but it is executed normally during the response phase. The cause of this transformation is the driving factors of the enterprise. Based on grounded theory analysis, the driving factor model for enterprise participation in emergency response in the context of digital cities is shown in Figure 3.

From Figure 3, the driving factors are mainly divided into three categories, namely environment, organization, and technology. In the context of the environment, driving factors involve the complexity and uncertainty of risks, modernization of emergency management, efficient emergency management needs of the public sector, and policies and regulations. In the organizational, driving factors include corporate reputation, self-development needs, participation ability, participation in practice, and social responsibility. In the technology, driving factors include data management capabilities, technological comparative advantages, intelligent emergency resource management, data disclosure, and new technologies. The driving factor model adopts a one-stage approach to complete the theoretical saturation test. Interpreting structural modeling is a widely used analytical method in modern systems engineering and a technique of structural modeling. The theoretical basis of TISM is to explain structural models. A system must have a structure, and the structure of the system determines its functionality, which is also the theoretical basis of TISM. The construction steps of TISM are divided into nine steps, including clarifying factors and variables, finding contextual relationships between factors, explaining the interrelationships between factors, interpreting pairwise comparison logic, establishing reachable matrices, obtaining reachable and antecedent sets for each factor, developing charts and drawing directed links, developing interaction matrices, and constructing the final model. The TISM model does not involve parameter selection. This model is widely used in overall design, regional planning, technical evaluation, and system diagnosis, and has strong applicability. Interpreting structural models can simplify complex systems into easily understandable and analytical graphics and matrices, helping us better understand the internal structure and dynamic behavior of the system (19–21). Therefore, the advantage of this model in this article is that it can better understand the driving factors of the enterprise. However, the limitation of the TISM model is that it requires high data requirements and is easily influenced by subjective factors. Public health events are often cross-regional and cross-sectoral, and it is difficult for a single agency or department to respond independently. Through collaborative governance, the resources and expertise of different departments and agencies can be pooled to jointly develop and implement response measures, thereby improving the efficiency and effectiveness of response to public health events. The impact of public health events is multifaceted, including health, social, economic and other aspects. Comprehensive collaborative governance enables more comprehensive identification and assessment of risks, ensuring effective prevention and response at all levels. In general, a model that combines collaborative governance of public health responses with full coverage of all public health events can improve the overall effectiveness of public health responses, better protect public health, and provide a solid foundation for addressing future challenges. The initial reachable matrix is shown in Equation (1) (22).


(1) $$A+B={\left(A+B\right)}^{n}={\left(A+B\right)}^{n+1}=R$$

**Figure 4:**
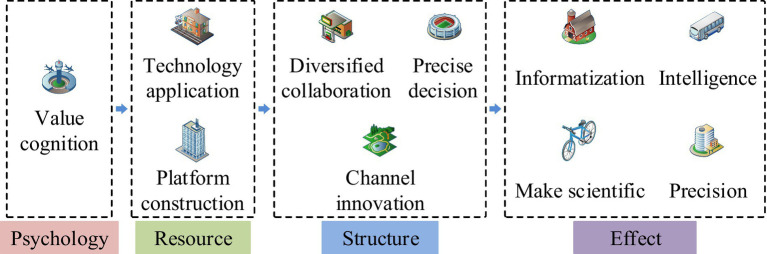
Empowerment mechanism for enterprises to participate in emergency response.

In Equation (1), A represents adjacency matrix. n represents the number of matrix calculations. B is the identity matrix. R represents the reachable matrix. To clarify the relationship between influencing factors, an expert scoring method is adopted. The final self interaction matrix is constructed under the principle of minority obeying majority. Considering the transitivity rule, the self interaction matrix is transformed into a reachable matrix. To analyze the driving force dependency between driving factors, the MICMAC method is used. The concept of MICMAC is a method of identifying variables with strong driving forces and prominent dependencies in complex systems by analyzing the driving and dependency relationships between factors. MICMAC is mainly composed of reachability matrix and driving force dependency matrix. The theoretical basis of the MICMAC model is the principle of matrix multiplication, and its construction process is divided into three steps. The first step is to clarify the driving force and dependency indicators, the second step is to divide the cluster, and the third step is to explain the cluster data. The MICMAC model does not have any parameters that need to be set. This model has been applied in innovative development, urban governance, and green construction, and has strong applicability. The advantage of the MICMAC method is that it can consider the mutual influence, degree, and direction between events, and can also systematically organize data with a large number of results into a form that is easy to analyze (23). The MICMAC method is mainly used to analyze the influence and attachment relationship between various factors in the system, reveal the mechanism of action between the influencing factors, and its results can be represented by coordinate axes (24). The advantage of this model in this article is its ability to analyze the impact and attachment relationships between enterprise driving factors, but its limitation is that it does not handle the complexity of the problem well enough. After using the MICMAC model for analysis, it is necessary to divide all driving factors into four clusters in quadrant form. In addition, the sum of the elements in each row of the reachability matrix constitutes the driving force of MICMAC analysis, and the sum of the elements in each column constitutes the dependency of MICMAC analysis. The grounded theoretical is also adopted to analyze the driving effectiveness of enterprise participation in public health emergency response. The driving effectiveness analysis based on grounded theory has three steps. The first is to collect relevant data. The second is to encode the data for the case text. The third step is to conduct a performance analysis. The data encoding in driving efficiency analysis also includes open, spindle, and selective encoding. The enabling mechanism for enterprises to participate in emergency response is shown in Figure 4.

From Figure 4, the empowerment mechanism for enterprises to participate in public health emergency response is divided into three empowerment modules, namely psychological, resource, and structural empowerment. Among them, psychological empowerment, as a condition for the empowerment mechanism, mainly involves the value perception of enterprises. Resource empowerment, as the foundation of the empowerment mechanism, mainly includes technology application and platform construction. Structural empowerment is the process of empowering mechanisms, mainly covering diverse collaboration, channel innovation, and precise decision-making. The effectiveness of the empowerment mechanism is mainly reflected in four aspects, namely informatization, intelligence, scientificity, and precision. The empowerment mechanism for enterprises to participate in public health emergency response also requires theoretical saturation testing.

### 3.2 Analysis method for spatiotemporal distribution characteristics of public health events oriented toward collaborative governance

In order to enhance the government’s emergency management capabilities for public health emergencies, the study analyzed from two perspectives: one is to compensate for the government’s shortcomings in emergency response from the perspective of enterprise entities, and the other is to enhance the government’s collaborative governance capabilities from the perspective of the spatiotemporal distribution characteristics of public health emergencies. In the previous chapter, a method design was conducted to investigate the driving factors of enterprise participation in public health emergencies in the context of digital cities. In this chapter, the research will design methods for optimizing government collaborative governance through spatiotemporal analysis in the context of digital cities. In the context of digitalization, both enterprises and governments should transform their previous thinking on responding to emergencies, pay more attention to the participation of digital technology, and promote the modernization and intelligence of emergency management. Sudden public health emergencies have strong uncertainty and diversity, which brings great challenges and difficulties to the emergency response of local governments. Therefore, in the modernization of emergency management, in addition to compensating for the government’s shortcomings in emergency management through enterprise participation, it can also enhance the government’s collaborative governance capabilities. From a management perspective, collaboration, as a close collaborative mechanism, can achieve common goals and tasks through collaboration between different units, departments, and organizations. Collaborative governance is an important responsibility of the government and relevant participating units in the face of sudden public health emergencies. The primary characteristic of collaborative governance is the diversification of governance entities, including organizations, institutions, and individuals who play important roles in governance. The characteristic of collaborative governance can be said to be a “center divergence” type, where “center” refers to the role of the government as the core in coordinating and taking overall responsibilities, and “divergence” refers to the process in which the government empowers other entities participating in governance. In addition, the characteristic of holistic governance is the “hourglass type,” where the upper end of the hourglass represents the overall government and the lower end represents other governance participants guided by the overall government (25). In intergovernmental collaborative governance, intergovernmental relations refer to the interlocking relationships formed by different governments in the vertical and horizontal directions, as well as the relationships between governments of different administrative divisions. Inter governmental relations can be divided into vertical and horizontal relationships, among which vertical inter governmental relations are divided into three types: central government and local government, superior government and subordinate government, and superior government and subordinate administrative departments. Horizontal intergovernmental relations refer to the relationships between different administrative divisions, governments without hierarchical affiliations, and various departments within the government (26). The intergovernmental relationship in collaborative governance discussed by the research institute is mainly horizontal, breaking the regional emergency management plan of a single local government. Therefore, it is necessary to coordinate and plan the emergency management plans of adjacent local governments based on the spatiotemporal distribution of sudden PHE, breaking the limitations of regional plans. In addition, based on the driving factors of enterprise participation in sudden PHE, the government can provide corresponding policy convenience to enterprises, thereby filling the shortcomings in government emergency response and achieving collaborative governance between the government and enterprise entities.

To better enhance the collaborative governance capabilities, the spatial autocorrelation analysis and spatiotemporal scanning statistics are adopted to analyze the distribution characteristics of sudden public health events in time and space, which serve as an important basis for the government to carry out collaborative governance. The development trend of PHE is gradually moving toward boundaryless, that is, crossing the boundaries of time and space, exceeding the administrative boundaries of local governments, which brings difficulties to the traditional governance model of regional local government units. Through spatial correlation analysis, it can provide data support for the government’s collaborative governance in PHE, clarify the distribution and diffusion characteristics of PHE, and enhance the government’s emergency response capability. Based on spatial econometric models, it is possible to analyze the influencing factors of PHE, which can help the government better understand the panel data of PHE, and based on this, formulate corresponding collaborative governance strategies to effectively respond to PHE. Spatial autocorrelation represents the interrelationships in space, mainly divided into three models, with distributions of positive correlation, negative correlation, and uncorrelation (27). For spatial autocorrelation analysis, the Moran’s I index is used for research and presented in ArcGIS software. The global Moran’s I index is displayed in Equation (2) (28).


(2) $$I=\frac{m\sum_{i}\sum_{j}w_{ij}\left(x_{i}-\bar{x}\right)\left(x_{j}-\bar{x}\right)}{\left(\sum_{i}\sum_{j}w_{ij}\right)\sum_{i}{\left(x_{i}-\bar{x}\right)}^{2}},I\in \left[1,-1\right]$$

In Equation (2), m stands for the provinces studied. i stands for the i-th province. j is the neighboring province of the i-th province. $w_{ij}$ stands for the spatial weight matrix. $x_{i}$ is the observed value for the i-th province. $x_{j}$represents the observed value of neighboring province j for the i-th province. $\bar{x}$ represents the average of the observed values. For local autocorrelation analysis, the Local Moran’s I index is adopted. The calculation is shown in Equation (3) (29).


(3) $$\mathrm{Local}\;\mathrm{Moran}’s\;I=\frac{\left(x_{i}-\bar{x}\right)\sum_{j}w_{ij}\left(x_{j}-\bar{x}\right)}{\sum_{i}{\left(x_{i}-\bar{x}\right)}^{2}}$$

**Figure 5:**
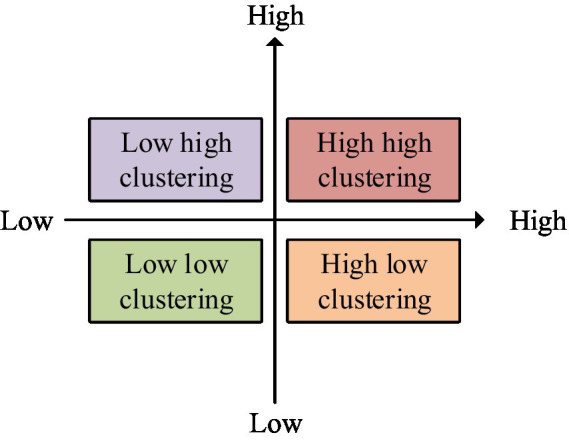
Four quadrants of local autocorrelation analysis.

For spatial matrices, inverse distance spatial matrice is used. Local autocorrelation analysis has four quadrants, namely high-high, high-low, low-high, and low-low. Different quadrants represent the different relationships between a phenomenon in a province and the neighboring provinces. The four quadrants of local autocorrelation analysis are shown in Figure 5 (30, 31).

In Figure 5, the first is the high-high altitude clustering. The second is low-high clustering. The third is low-low clustering. The fourth is high-low clustering. The first character in the four clusters represents the level of a phenomenon in a certain province or region. The second character represents the level of neighboring provinces in a certain phenomenon. For example, the first high in a high-high clustering represents a province with a higher level of phenomenon. The second high represents that adjacent provinces also have a higher level of phenomenon. The interpretation of other clusters can also be obtained in the same way. Through spatiotemporal scanning statistics, the aggregation status of data in both time and space can be analyzed. Therefore, SaTScan software is selected to complete the application of this method. The main task of spatiotemporal scanning statistics is to calculate expected observations based on actual observations. The logarithmic likelihood ratio test statistic is calculated based on actual and observed values. This statistic is shown in Equation (4) (32).


(4) $$LRR=\log\left(\frac{c}{\mu }\right){\left(\frac{C-c}{C-\mu }\right)}^{\left(C-c\right)}$$

In Equation (4), C represents the sum of actual observed values in each region. c represents the actual observation value within the scanning window. LRR is the logarithmic likelihood ratio test statistic. μ represents the expected value within the scanning window. The window with the highest LRR value is the high clustering window. The LRRvalue requires hypothesis testing and the significance level *P* is calculated. When *p* < 0.05, it indicates that the value has significant statistical characteristics. In addition, through spatiotemporal scanning statistical methods, the relative risk index can also be constructed. A high risk index value indicates a greater likelihood of actual value expansion. To better analyze the spatiotemporal distribution characteristics of PHE, interannual concentration index and variation coefficient methods are also used. The interannual concentration index is shown in Equation (5) (33).


(5) $$Y=\sqrt{\frac{\overset{h}{\underset{g=1}{\sum }}{\left(Z_{g}-\frac{1}{h}\right)}^{2}}{h}}$$

In Equation (5), Y represents the interannual concentration index. h represents the years included in a certain period of time. $Z_{g}$ represents the numerator value of the events in year g to the total events during this period. A higher Y-value indicates that the changes in events are more unstable. To measure the time concentration of PHE, the seasonal intensity index is used. The calculation is shown in Equation (6) (34).


(6) $$D=\sqrt{{\frac{\overset{12}{\underset{v}{\sum }}\left(q_{v}-8.33\right)}{12}}^{2}}$$

In Equation (6), D represents the seasonal intensity index. $q_{v}$ represents the ratio of the events in month v to the total events throughout the year. To study the distribution balance of PHE in different provinces, an imbalance index is used. The calculation is shown in Equation (7).


(7) $$S=\frac{100\overset{q}{\underset{o=1}{\sum }}T_{o}-50\left(q+1\right)}{100q-50\left(q+1\right)}$$

In Equation (7), S represents the imbalance index. q represents the number of PHE. The proportion of sudden public health incidents in various provinces in the total incidents nationwide is calculated. $T_{o}$ is the cumulative percentage of the o-th after sorting these proportions from highest to lowest. The difference in PHE between different provinces is measured by the variation coefficient, which is calculated as shown in Equation (8).


(8) $$CV=\frac{1}{q}\sqrt{\frac{\overset{m}{\underset{i=1}{\sum }}{\left(q_{i}-\bar{q}\right)}^{2}}{m-1}}$$

In Equation (8), CV represents the coefficient of variation. $q_{i}$ represents the number of public health emergencies in i province. $\bar{q}$ is the average value of PHE. To better analyze the spatial distribution characteristics of public health emergencies, the kernel density estimation method is also adopted. The calculation is shown in Equation (9) (35).


(9) $$f\left(\alpha \right)=\frac{1}{m\beta }\overset{m}{\underset{i=1}{\sum }}K\left(\frac{\alpha -\alpha_{i}}{\beta }\right)$$

In Equation (9), β represents the radius of the circular domain. α represents the valuation point. $K\left(\frac{\alpha -\alpha_{i}}{\beta }\right)$ is the kernel density function. $\alpha_{i}$ represents the event. $\left(\alpha -\alpha_{i}\right)$ is the distance from α to $\alpha_{i}$. Through the panel data model, the influencing factors of sudden public health events can be analyzed. The expression of this model is shown in Equation (10).


(10) $$\phi_{i\varepsilon }=\Pi_{i}+{ϒ}_{i\varepsilon }\Gamma_{i\varepsilon }+\delta_{i\varepsilon }$$

In Equation (10), Π is a constant. ϒ is the coefficient. ε represents the year. $\phi_{i\varepsilon }$ stands for the dependent variable. $\Gamma_{i\varepsilon }$ stands for the explanatory variable. $\delta_{i\varepsilon }$ is the random error term of the cross-sectional individual over time. Panel data needs to be verified. The test statistic is shown in Equation (11) (36).


(11) $$F=\frac{\left(\partial_{2}-\partial_{1}\right)/\left[\left(\varphi -1\right)\left(\gamma +1\right)\right]}{\partial_{1}\left[\varphi \eta -\varphi \left(\gamma +1\right)\right]}$$

**Figure 6:**
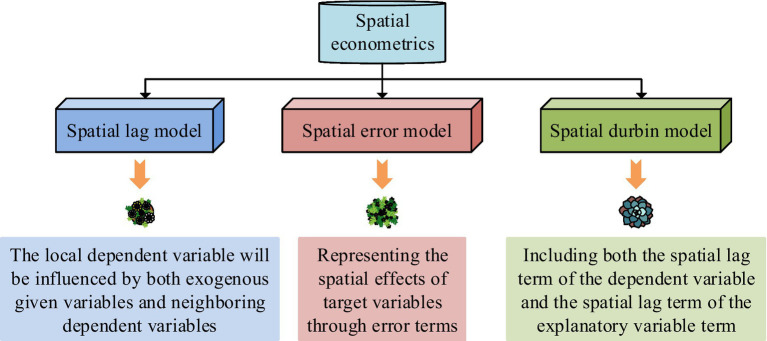
Common spatial econometric models.

In Equation (11), $\partial_{1}$ represents the residual bisection sum of the random effects model. $\partial_{2}$ represents the residual bisection sum of the mixed effects model. φ is the sections. φ represents the explanatory variables. η is the number of periods. However, traditional panel data models lack consideration for geographic space. Therefore, a panel space econometric model is applied to analyze the influencing factors of public health events. There are three common types of spatial econometric models, as shown in Figure 6 (37).

From Figure 6, common spatial econometric models include spatial lag model, spatial error model, and spatial Durbin model. The spatial lag model assumes that the local dependent variable will be influenced by both exogenous given variables and neighboring dependent variables. The assumption of the spatial error model is to represent the spatial effects of the target variable through the error term. The spatial Durbin model solves the variable omission in the spatial lag model and spatial error model. The spatial lag model is displayed in Equation (12) (38).


(12) $$\lambda_{\theta \tau }=\rho w_{ij}\lambda_{\theta \tau }+\sigma \psi_{\theta \tau }+\zeta_{\theta \tau }$$

In Equation (12), ρ represents the regression coefficient of spatial lag effect. σ stands for the estimated coefficient vector of the explanatory variable. $w_{ij}\lambda_{\theta \tau }$ is the spatial lag term. $\zeta_{\theta \tau }$ represents the random error term of the model. $\lambda_{\theta \tau }$ represents the observed value of the dependent variable in the spatial unit θ in year τ. $\psi_{\theta \tau }$ stands for the observed value of the explanatory variable for the spatial unit θ in year τ. The spatial error model is shown in Equation (13) (39).


(13) $$\lambda_{\theta \tau }=\sigma \psi_{\theta \tau }+\Theta_{\theta \tau }$$

In Equation (13), $\Theta_{\theta \tau }$ represents the random error term of the spatial error model. The calculation is shown in Equation (14).


(14) $$\Theta_{\theta \tau }=ƛw_{ij}\Theta_{\theta \tau }+\zeta_{\theta \tau }$$

In Equation (14), ƛ represents the regression coefficient of the spatial error effect. $w_{ij}\Theta_{\theta \tau }$ is the spatial lag value. The spatial Durbin model is shown in Equation (15) (40).


(15) $$\lambda_{\theta \tau }=\rho w_{ij}\lambda_{\theta \tau }+\sigma \psi_{\theta \tau }+ƛw_{ij}\psi_{\theta \tau }+P_{\tau }+\Lambda_{\theta }+\zeta_{\theta \tau }$$

**Figure 7:**
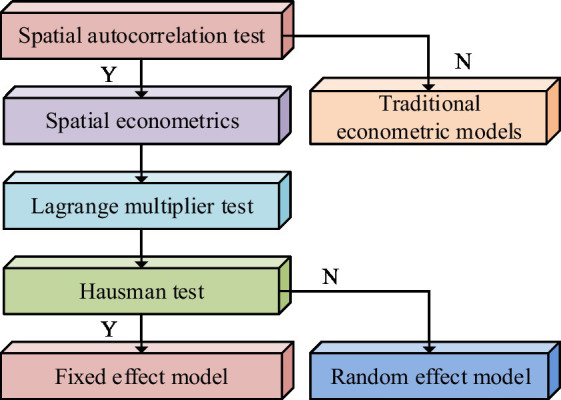
Discriminant steps for spatial econometric models.

In Equation (15), $\Lambda_{\theta }$ represents individual effects. $P_{\tau }$ represents the time effect. To select spatial econometric models, it is discriminated. The discrimination steps of the spatial econometric model are shown in Figure 7.

From Figure 7, the first in distinguishing the spatial econometric model is to use the global Moran’s I index to test the spatial autocorrelation. If the test passes, the spatial econometric model will be adopted. Otherwise, the traditional econometric model will be used. The second step is to use Lagrange multipliers to test the model. The model with higher significance is selected. The third step is to perform the Hausman test. If the test passes, a fixed effects model will be adopted. Otherwise, a random effects model will be used.

## 4 Optimization path of enterprise emergency response and government collaborative governance measures in the digital cities

In this chapter, some cases of enterprises participating in emergency response were selected. The hierarchical division results and driving force-dependency MICMAC results of enterprise participation in emergency response were analyzed. Based on this, the optimization path for enterprise participation in emergency response is analyzed. In addition, the spatial and temporal distribution characteristics of PHE were analyzed. Then the collaborative governance of the government was analyzed.

### 4.1 Optimization path for enterprises to participate in public health emergency response in the context of digital cities

The relationship and hierarchical structure between the driving factors of enterprise participation in emergency response were analyzed. Based on this, the optimization path for enterprise participation in emergency response was proposed. The 2020 major infectious pneumonia public health event and 15 cases of enterprises participating in emergency response were selected for analysis. The technology products of enterprises in the epidemic prevention were also selected. The data obtained in the analysis of driving factors and hierarchical structure in the study are qualitative data. In addition to the selected enterprise cases, it also involves government party media information public platforms, CNKI academic platforms, and news reports, and theoretical saturation tests were conducted on these materials. The construction results of the final matrix were displayed in Table 1.

**Table 1:** The construction result of the final reachable matrix.

Factor	A	B	C	D	E	F	G	H	I	J	K	L	M	N	Drive
A	1	1^#^	0	1^#^	0	1^#^	0	1	0	1^#^	1^#^	0	1	1	9*
B	0	0	0	0	0	1	0	0	0	1	0	1^#^	1^#^	0	4*
C	0	0	0	0	0	0	0	0	0	0	0	1	0	0	1*
D	0	0	0	0	1	0	0	0	0	0	0	0	0	0	1*
E	0	1^#^	0	1	1^#^	1^#^	1^#^	0	1^#^	0	0	1^#^	1	1	9*
F	1	1^#^	0	1^#^	0	1	0	1	0	1	1	1^#^	1	1^#^	10*
G	1	1^#^	1	1^#^	1	1	0	1	0	1	1	1	1	1^#^	12*
H	1^#^	0	0	0	0	1^#^	0	1	0	1	1	0	1^#^	0	6*
I	0	1^#^	0	1^#^	0	1	0	0	0	0	0	1^#^	1	0	5*
J	0	1	0	1	1^#^	1	1^#^	0	1^#^	0	1^#^	1^#^	1	0	9*
K	0	1	1^#^	1	1	1^#^	1	0	1	1^#^	1^#^	1	0	0	10*
L	0	1	0	0	1^#^	1^#^	0	1^#^	1	1	1	1	0	0	8*
M	1^#^	1	1	0	1	1^#^	1	1^#^	1	1^#^	1^#^	1^#^	1^#^	0	12*
N	0	1	0	0	1	1	0	1^#^	0	1^#^	1	1	1^#^	0	8*
Dependence	5*	10*	3*	7*	8*	12*	4*	7*	5*	9*	9*	11*	10*	4*	/

^#^indicated transit between driving factors, and *represented *p* <0.05, which indicates that the data has statistical significance.

**Figure 8:**
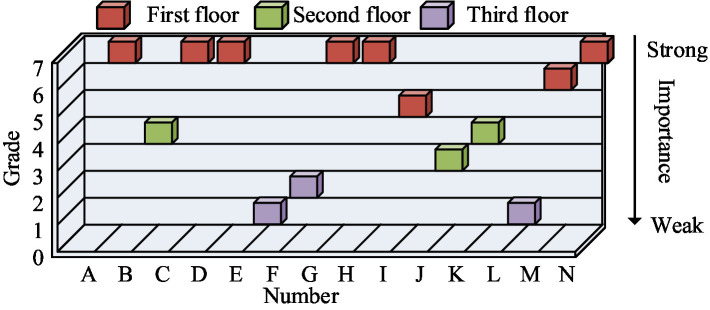
Hierarchy of driving factors for enterprise participation in emergency response results.

In Table 1, 1 represented a direct relationship between driving factors. 1^#^ indicated transitivity between driving factors. 0 represented that there is no relationship between the driving factors. * representing *p* < 0.05, this indicates that the data has statistical significance. A was data management capability. B represented policies and regulations. C represented the development needs of the enterprise itself. D represented the complexity and uncertainty of the risk. E represented data disclosure. F represented the participation of enterprises in practice. G was the demand for efficient emergency management in the public sector. H represented the reputation of the enterprise. I was the modernization of emergency management. J represented intelligent emergency resource management. K represented corporate social responsibility. L represented a new technology. M represented the ability of the enterprise to participate. N represented the relative advantage of technology. On the basis of the final reachable matrix, the driving factors for all enterprises to participate in emergency response were hierarchically divided. The partitioning results were shown in Figure 8.

**Figure 9:**
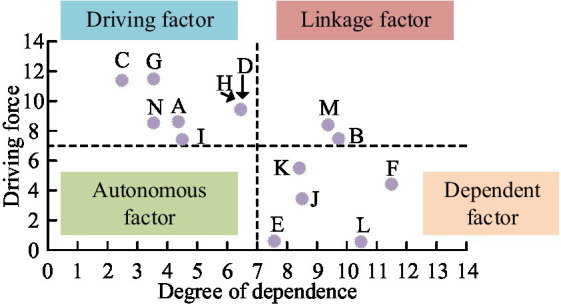
MICMAC analysis of driving force dependency among driving factors.

From Figure 8, the hierarchical structure had three layers. The first layer involved three levels, from level 5 to level 7. The second layer involved two levels, namely Level 3 and Level 4. The third layer also involved two levels, namely Level 1 and Level 2. In the hierarchical structure of the TISM model, the most important factor was located at the first level of the hierarchy. The importance of factors decreased sequentially from the first level to the third level. Among the driving factors for enterprises to participate in public emergency response, the most important factors included the development needs of enterprises themselves, modernization of emergency management, relative technological advantages, and the need for efficient emergency management in the public sector. These factors served as optimization paths for enterprises to participate in emergency response. Enterprises could optimize and improve key driving factors by analyzing their problems in reality, especially the optimization of digital technology. The driving force-dependency MICMAC analysis between driving factors was shown in Figure 9.

From Figure 9, in the MICMAC analysis of driving force-dependency, the driving factors were divided into four groups according to the strength of driving force-dependency. The driving factors within the upper left corner were high driving force and low dependency. There were a total of 7 driving factors in this group, mainly involving data management capabilities, technological comparative advantages, etc. The factors in the lower left corner range had low dependence and driving force. There were no driving factors in this group. The factors within the upper right corner were high driving force and high dependency, with a total of 2 driving factors in this group. The factors in the lower right corner were low driving force and high dependency. There were a total of 5 driving factors in this group. The driving factors of high driving force and low dependence were the most important factors. Therefore, for enterprises, technology and data could be used as optimization paths. Through open coding, spindle coding, selective coding, and theoretical saturation testing, the driving effectiveness of enterprise participation in public health emergency response involved psychological empowerment, resource empowerment, and structural empowerment.

### 4.2 Government collaborative governance strategies based on temporal and spatial distribution characteristics

**Figure 10:**
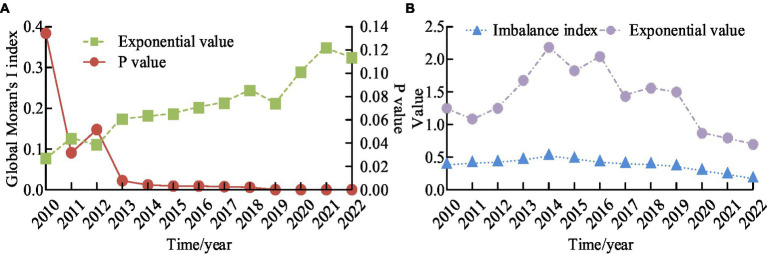
Trends in the global Moran’s I index, imbalance index, and coefficient of variation of public health emergencies from 2010 to 2022. **(A)** Trend chart of Moran’s I index values and *P-*values over the years. **(B)** The trend of changes in imbalance index and coefficient of variation.

To analyze the spatiotemporal distribution characteristics of PHE, detailed information on PHE in 31 provinces, municipalities, and autonomous regions (excluding Hong Kong, Macau, and Taiwan) from 2010 to 2022 was selected for analysis. The obtained data information of PHE was preprocessed. Multi channel data queries were used to fill in missing values and classify, summarize, and organize data. In the analysis of spatiotemporal distribution characteristics, the selected samples for research are mainly quantitative data, such as infection rates, the number of public health emergencies, and the number of provinces. The global Moran’s I index, imbalance index, and variation coefficient of PHE from 2010 to 2022 were shown in Figure 10.

**Figure 11:**
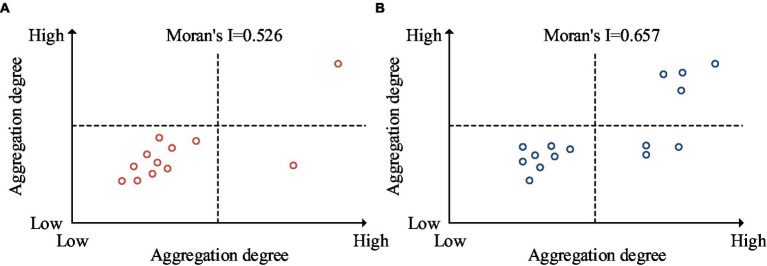
Local spatial aggregation results of public health emergencies in 2018 and 2021. **(A)** 2018 local spatial aggregation results. **(B)** 2021 local spatial aggregation results.

From Figure 10A, as the year increased, the global Moran’s I index also displayed a gradually increasing trend overall. The maximum value was 0.36, which occurred in 2021. The minimum value was 0.075, which occurred in 2010. The maximum value of *P* was 0.136, which occurred in 2010. The minimum value was 0, which occurred in 2019 and continued until 2022. The change in *p*-value decreased with the increase of years. From Figure 10B, the maximum value of the imbalance index was 0.51 and the minimum value was 0.25. As the years increased, the imbalance index showed a trend of gradually increasing and then decreasing. The maximum value of the variation coefficient was 2.21, and the minimum value was 0.75. Overall, it showed a trend of first increasing and then decreasing. There was a positive spatial correlation between PHE in China from 2010 to 2022. The spatial clustering phenomenon in PHE in 2011 and 2017 was relatively obvious. After 2019, the spatial aggregation degree of public health emergencies had rapidly increased. There was no random situation. The local spatial aggregation results of PHE in 2018 and 2021 were shown in Figure 11.

**Figure 12:**
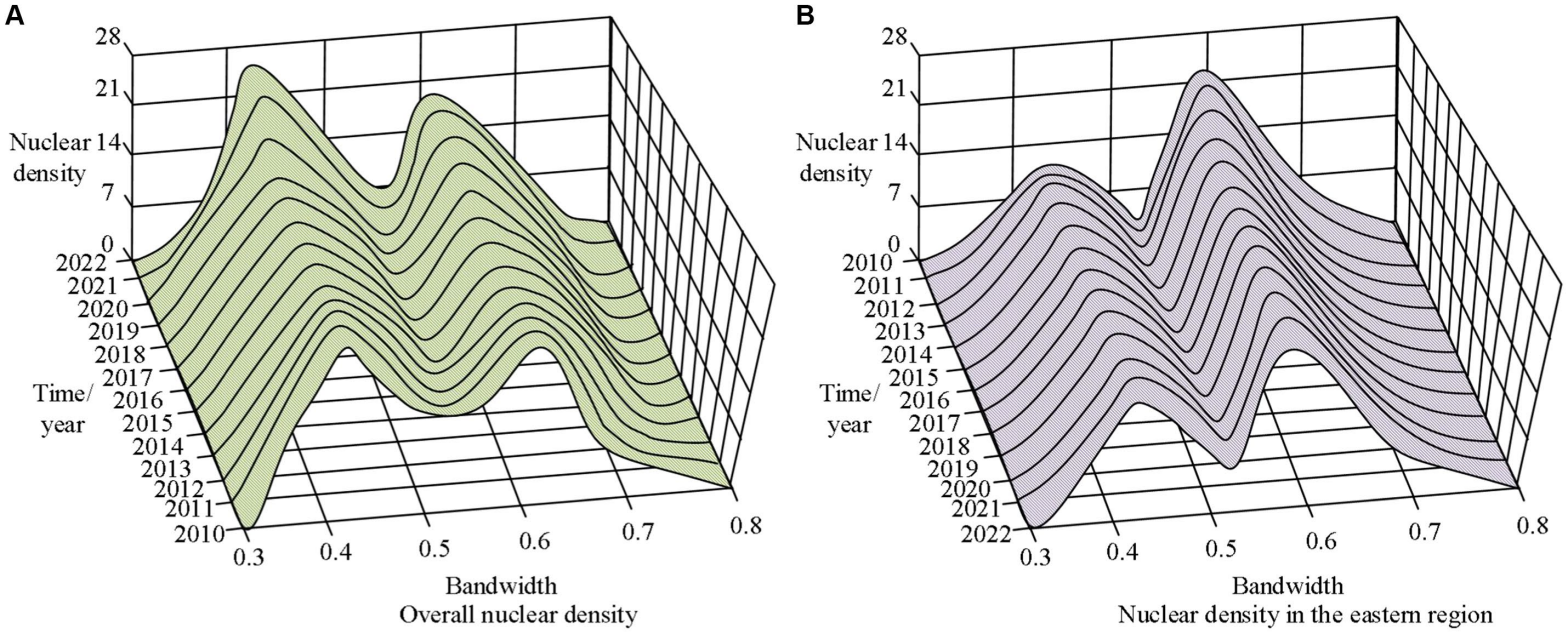
Nuclear density analysis of public health emergencies. **(A)** Overall nuclear density. **(B)** Nuclear density in the eastern region.

From Figure 11A, the local Moran’s I value for 2018 was 0.526. The region with high-high clustering was Tibet. The region with high-low clustering was Inner Mongolia. There were currently no areas with low-high clustering. The regions with low-low clustering include Jilin, Liaoning, Hebei, Beijing, Tianjin, Shandong, Anhui, Jiangsu, Shanxi, and Henan. In Figure 11B, the local Moran’s I value for 2021 was 0.657. The regions with high-high clustering included Tibet, Guangxi, Guangdong and Hainan. The regions with high-low clustering included Inner Mongolia, Shanxi, and Anhui. There were currently no areas with low-high clustering. The regions with low-low clustering were Jilin, Liaoning, Hebei, Beijing, Tianjin, Shandong, Henan, and Jiangsu. There were differences in the types of local spatial aggregation between 2018 and 2021. This also indicated that the distribution of PHE in China was uneven among provinces. The nuclear density analysis of PHE in China was displayed in Figure 12.

**Figure 13:**
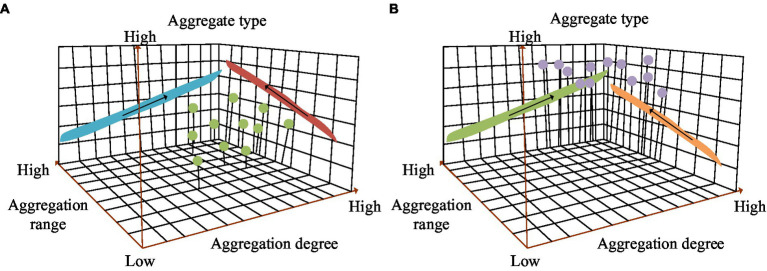
Spatial and temporal clustering characteristics of public health emergencies in different regions from 2010 to 2022. **(A)** Characteristics of spatiotemporal aggregation in the eastern region. **(B)** Characteristics of spatiotemporal aggregation in the western region.

From Figure 12A, the maximum value of overall nuclear density in China was 26.1246, while the minimum value was 0. As the years increase, the maximum value curve begins to shift to the left. In Figure 12B, the maximum value in the eastern region of China was 24.7781, while the minimum value was 0. In addition, the peak of nuclear density in the eastern region gradually decreased with the increase of years. The overall level of PHE in the western region of China exceeded other regions. The spatiotemporal clustering characteristics of PHE in different regions from 2010 to 2022 were shown in Figure 13.

**Figure 14:**
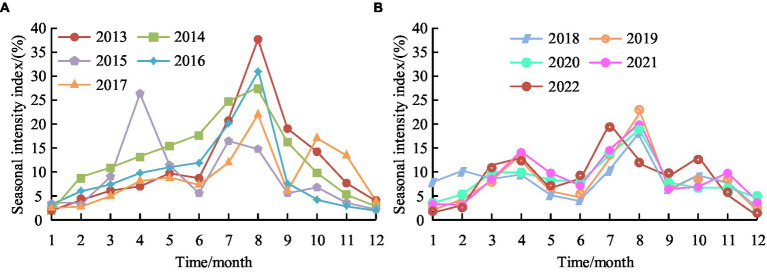
Seasonal intensity index results of public health emergencies. **(A)** Seasonal intensity index from 2013 to 2017. **(B)** Seasonal intensity index from 2018 to 2022.

In Figure 13A, the aggregation type in the eastern region of China was low value aggregation. As the years increased, the aggregation degree gradually increased. The gathering range was also gradually expanding. From Figure 13B, the aggregation type in the western region of China was high value aggregation. The aggregation degree was also increasing year by year. The gathering range also gradually expanded with the increase of years. The clustering type results of spatiotemporal scanning analysis were roughly consistent with those of spatial autocorrelation analysis. Based on the spatial distribution characteristics of PHE, local governments in China can make efforts to coordinate and manage them at the spatial level to avoid the spread and overflow of PHE. In addition, local governments can establish different types of regional collaborative governance models, fully considering the spatial effects of sudden public health events, and improving their emergency response capabilities. The seasonal intensity index results of PHE were displayed in Figure 14.

From Figure 14A, the maximum value of the seasonal intensity index in 2013 was 36.91% in August, and the minimum value was 1.12% in January. The maximum value of the seasonal intensity index in 2014 was 27.21% in August, and the minimum value was 1.12% in January. The maximum value of the seasonal intensity index in 2015 was 25.88% in April, and the minimum value was 2.88% in January. The maximum value of the seasonal intensity index in 2016 was 30.36% in August, and the minimum value was 2.21% in January and February. The maximum value of the seasonal intensity index in 2017 was 22.29% in August, and the minimum value was 1.67% in January. From Figure 14B, the maximum values of the seasonal intensity index from 2018 to 2021 were in August, which were 17.96, 22.99, 18.18, and 18.76%, respectively, while the minimum values were 3.48, 2.81, 3.94, and 3.16%, respectively. The maximum value of the seasonal intensity index in 2022 was 19.75% in July, and the minimum value was 0.91% in December. Based on the periodicity and periodicity of PHE in time, local governments can implement different collaborative governance tasks at different stages of PHE, further improving their emergency response capabilities and promoting the modernization of emergency management.

## 5 Discussion

In order to enhance the government’s ability in PHE emergency response, research was conducted from two aspects: emergency entities and collaborative governance. The study explores the driving factors of corporate entities as social forces participating in emergency response, and analyzes the spatiotemporal distribution characteristics of PHE as a basis for collaborative governance. The results show that the key driving factors for enterprises to participate in emergency response include their own development needs, relative technological advantages, efficient emergency management needs of the public sector, and modernization of emergency management. Psychological empowerment focuses on improving objective external conditions (such as institutions, society, economy, politics, and environment), empowering individuals with the power to take action. By analyzing key driving factors, it is possible to identify the power that empowers the subject to take action. Under the technological advantage, enterprises can utilize digital technology to develop products and drive their participation in emergency response, such as the emergency material supply chain big data management platform of China JD Logistics and the intelligent epidemic prevention black technology matrix created by SAIC GM Wuling. In order to understand the main driving factors affecting the changes in the water body of Poyang Lake, Zhu et al. (41) analyzed the spatiotemporal changes of the four seasons of Poyang Lake through 124 remote sensing images, and found that both human and natural factors are the main driving factors affecting the changes in the water body of the lake. It can be seen that the results of the study are similar to those of experts such as Zhu et al. (41). In addition, the western region of China has the highest level of PHE, with the strongest seasonal intensity index from 2013 to August 2022. The collaborative governance theory believes that the internal elements and modules of the system can improve the overall operational efficiency through mutual cooperation. For example, the joint prevention and control mechanism adopted by Shanghai, China in the COVID-19 epidemic has ensured the parallel disposal and orderly response of different departments, and improved the overall operational efficiency of the system. Ray et al. (42) conducted a retrospective study based on real data to analyze the spatiotemporal clustering between opioid drug seizures and excessive drug demand in surrounding geographical areas. They found a significant correlation between opioid drug seizures and an increase in spatiotemporal clustering in the demand for seeking help from excessive drugs in nearby areas (42). The results of the study are somewhat similar to those of researchers such as Ray et al. (42).

During the COVID-19 epidemic, the Chinese government closely followed the changes of the epidemic to update its prevention and control measures. The name of novel coronavirus pneumonia was changed to novel coronavirus infection, and then it was officially adjusted from “Class A management measures for Class B infectious diseases” to “Class B management measures for Class B infectious diseases.” The Chinese government made scientific decisions according to the time and situation. In addition, Chinese enterprises also played their role as social forces during the COVID-19. For example, retail enterprise Hema Xiansheng used digital systems to pick, transmit and pack goods. Tencent and Ali developed health codes under the guidance of the government. The participation of these corporate social forces has filled the gap in the government’s emergency management capabilities.

Drugs and drug management are important components of public health emergency response. The government should develop emergency plans to respond to the supply and management of drugs under PHE, such as setting up relevant management teams, ensuring the reserve, supply, and normal operation of emergency drugs and other materials. In addition, enterprises can also assist the government in emergency response to drugs and drug management, such as pharmaceutical companies borrowing drugs and drugs from the government and handling procedures afterwards.

There are also certain shortcomings and limitations in the research. Firstly, the influencing factors of the research formulation are not detailed enough. Therefore, future research can expand the scope of case selection and select representative cases based on the type of unexpected event. Afterwards, we will code the influencing factors and divide them into levels to provide a basis for decision-makers. Secondly, the social forces selected for the study are only corporate entities. Future research can increase the analysis of other participating entities, such as volunteers, social rescue teams, charitable organizations, communities, social organizations, etc., and increase the types and quantities of case studies for driving factor analysis. Thirdly, the spatiotemporal analysis of PHE mainly focuses on the macro level. Future research can expand the scope of data collection and place the research area in smaller administrative units (such as prefecture level cities and towns) and time scales (such as days and hours). Fourthly, the collaborative governance model and content mainly focus on the theoretical level. Therefore, future research needs to start from a practical perspective, conduct research on the emergency management capabilities and economic conditions of governments in various parts of China, and combine theory and practice.

## 6 Conclusion

To make up for the shortcomings of the government in emergency response to PHE, the driving factors and driving effectiveness of enterprise participation in emergency response are analyzed based on grounded theory. Based on spatial autocorrelation analysis and spatiotemporal scanning statistics, the spatiotemporal characteristics of PHE are analyzed. Based on this analysis, the government’s collaborative governance strategies are explored. The research results show that there are 14 driving factors for enterprises to participate in emergency response. The most important is the own development needs and relative technological advantages of the enterprise itself. Therefore, enterprises should actively utilize the advantages of digital technology in the context of digitalization to enhance their technological advantages. The combination of government power and technological advantages of enterprises can enhance the resilience of digital cities under PHE. Under PHE, digital cities not only have the institutional advantages of the Chinese government, people-oriented humanistic care support, and strong material and technological guarantees, but also have the support provided by enterprises in terms of technology. The maximum value of the global Moran’s I index was 0.36, which occurred in 2021. The minimum value was 0.075, which occurred in 2010. The maximum value of the imbalance index was 0.51, and the minimum value was 0.25. The maximum value of the variation coefficient was 2.21, and the minimum value was 0.75. There was a positive spatial correlation between PHE in China from 2010 to 2022. From 2010 to 2011, the maximum value of overall nuclear density in China was 26.1246, while the minimum value was 0. The maximum value in the eastern region of China was 24.7781, and the minimum value was 0. The overall level of PHE in the western region exceeded other regions. The maximum value of the seasonal intensity index in the past decade was mainly concentrated in the range of 10 to 40%, and the minimum value was in the range of 1 to 4%. In addition, PHE has mainly occurred in August in the past decade. The spatiotemporal distribution characteristics of PHE can provide data support for government emergency response and enterprise emergency participation. It can be concluded from the experimental results that the PHE collaborative governance theory based on the rooted theory can improve the decision-making process and make it more transparent and inclusive. Through collective wisdom and the participation of multiple stakeholders, more comprehensive and effective public health strategies can be developed. Collaborative governance theory promotes the effectiveness of risk and crisis management. When multiple parties are involved, potential risks can be identified more quickly, preventive measures can be taken, and a rapid response can be made when a crisis occurs. Both grounded theory and collaborative governance theory are highly effective theories when detached from real-life scenarios. The grounded theory itself is scientific, rigorous, effective, and legitimate, with strong universality, and can be applied to research problems that require in-depth exploration and analysis. Collaborative governance theory is an effective social governance approach that has been widely applied in societies around the world and has strong universality. The spatiotemporal distribution characteristics can only be analyzed after the occurrence of PHE. By analyzing the spatiotemporal distribution characteristics of similar events and rehearsing the government’s collaborative governance strategies, it can provide empirical support for subsequent PHE emergency response, enrich the government’s governance experience in PHE, and improve the emergency response capability of digital cities.

## Data availability statement

The original contributions presented in the study are included in the article/supplementary material, further inquiries can be directed to the corresponding author.

## Author contributions

YC: Investigation, Supervision, Writing – review & editing. YY: Investigation, Writing – original draft.
